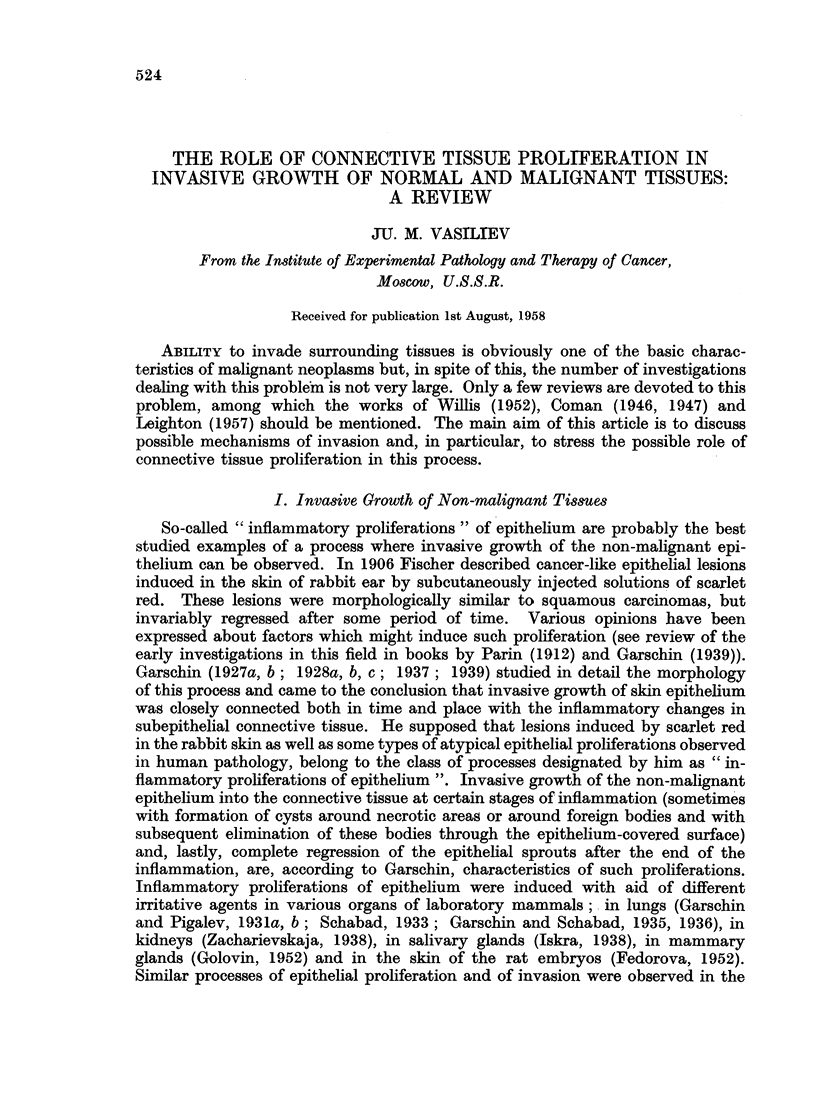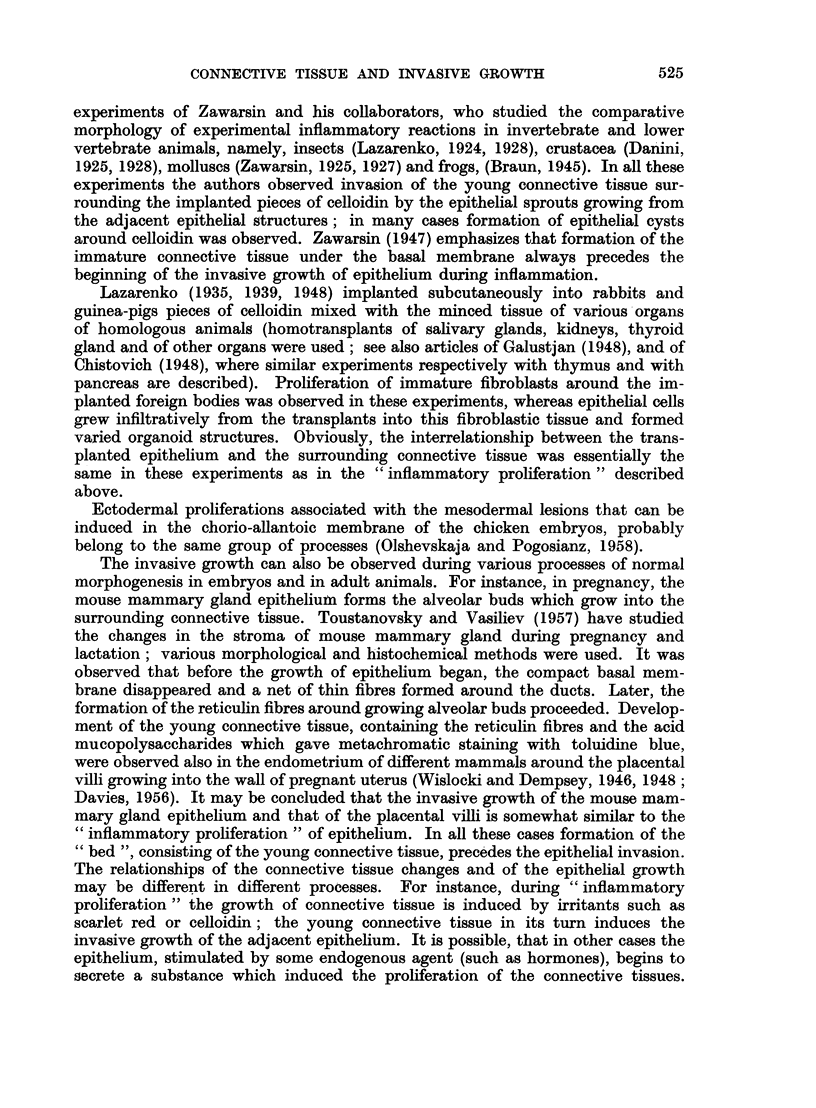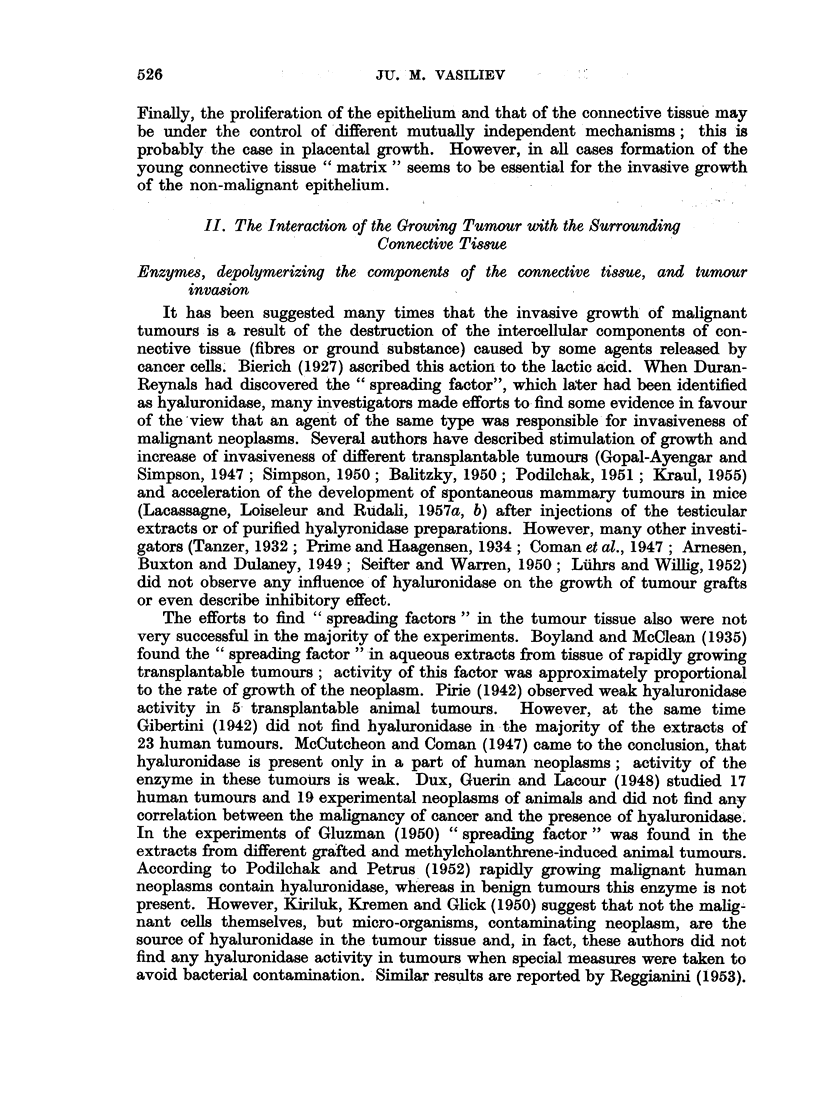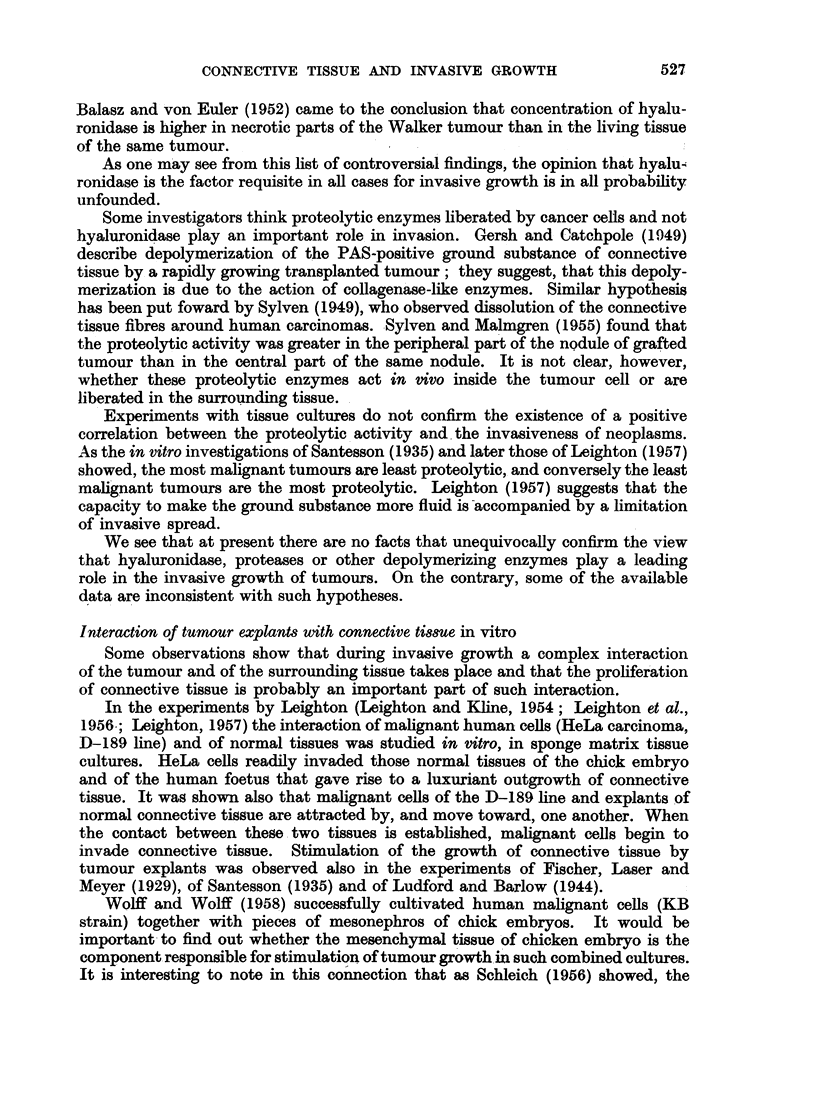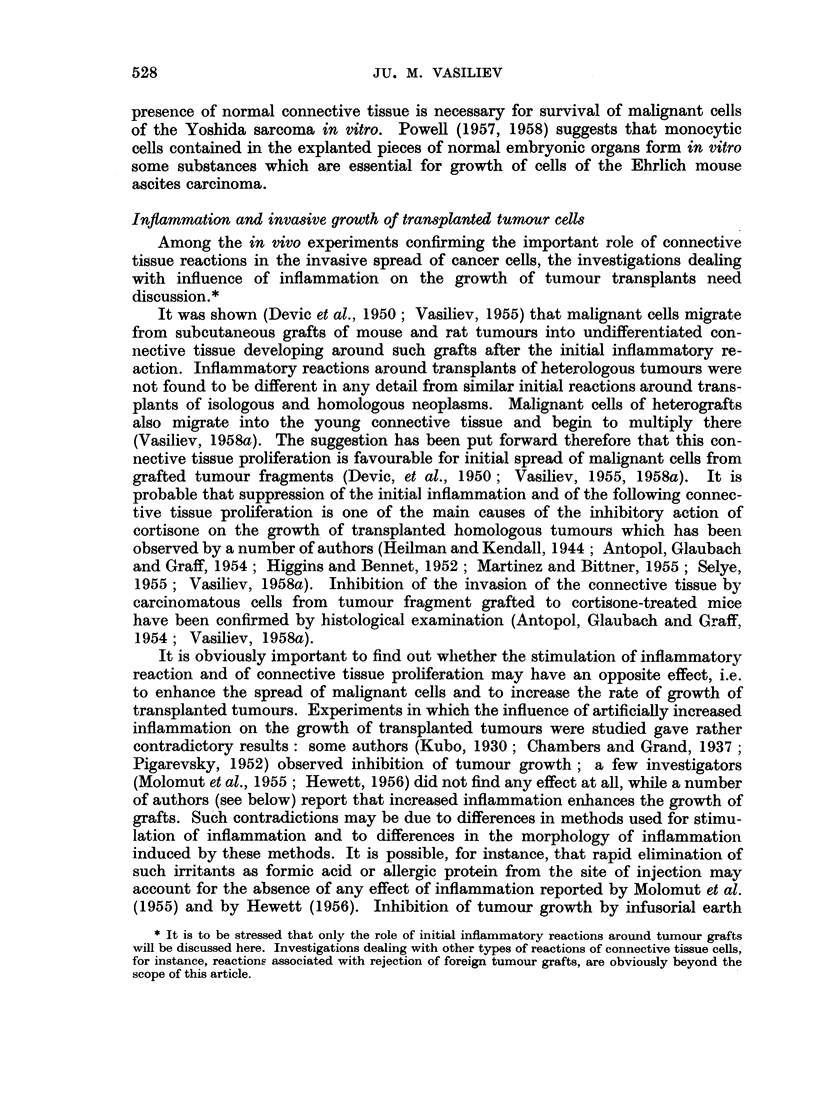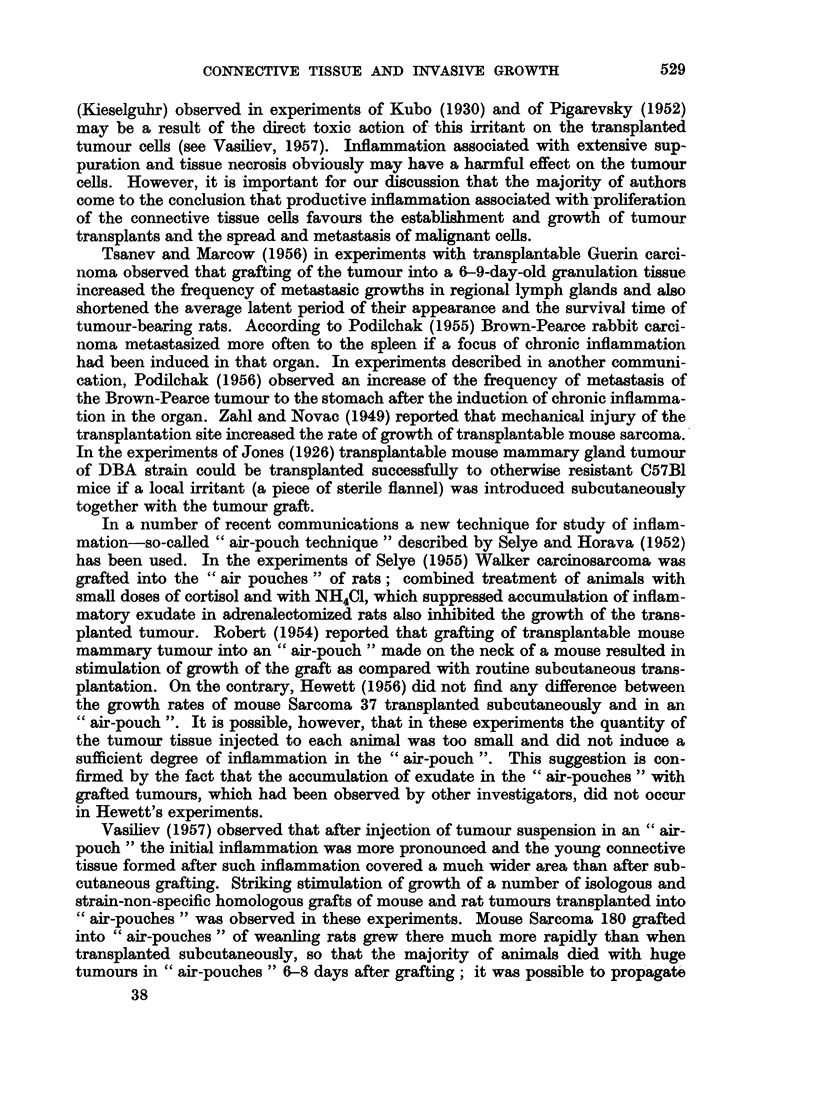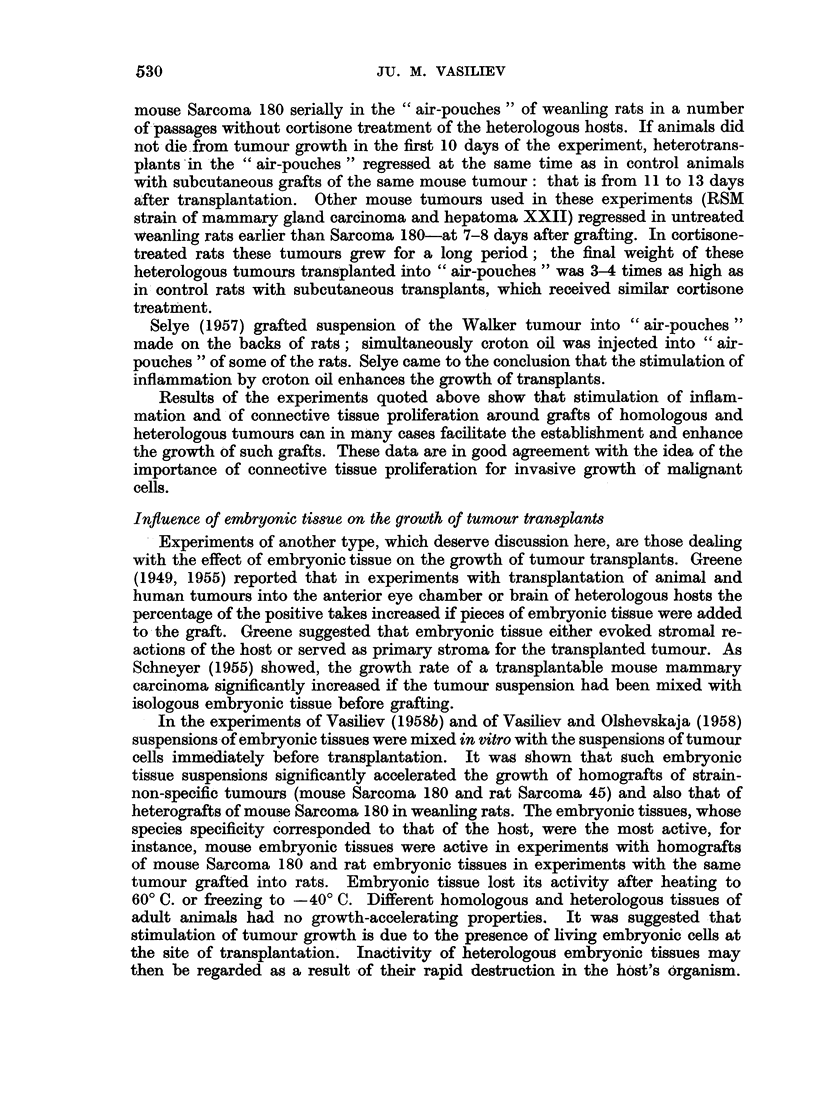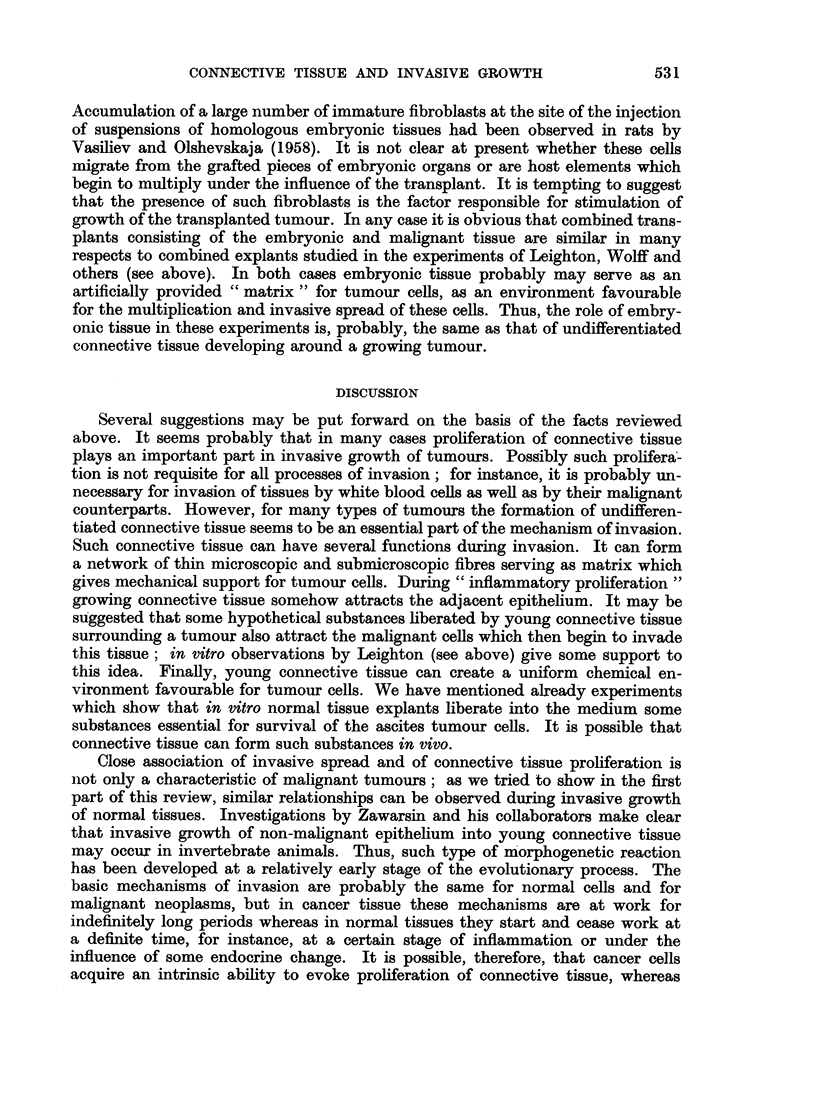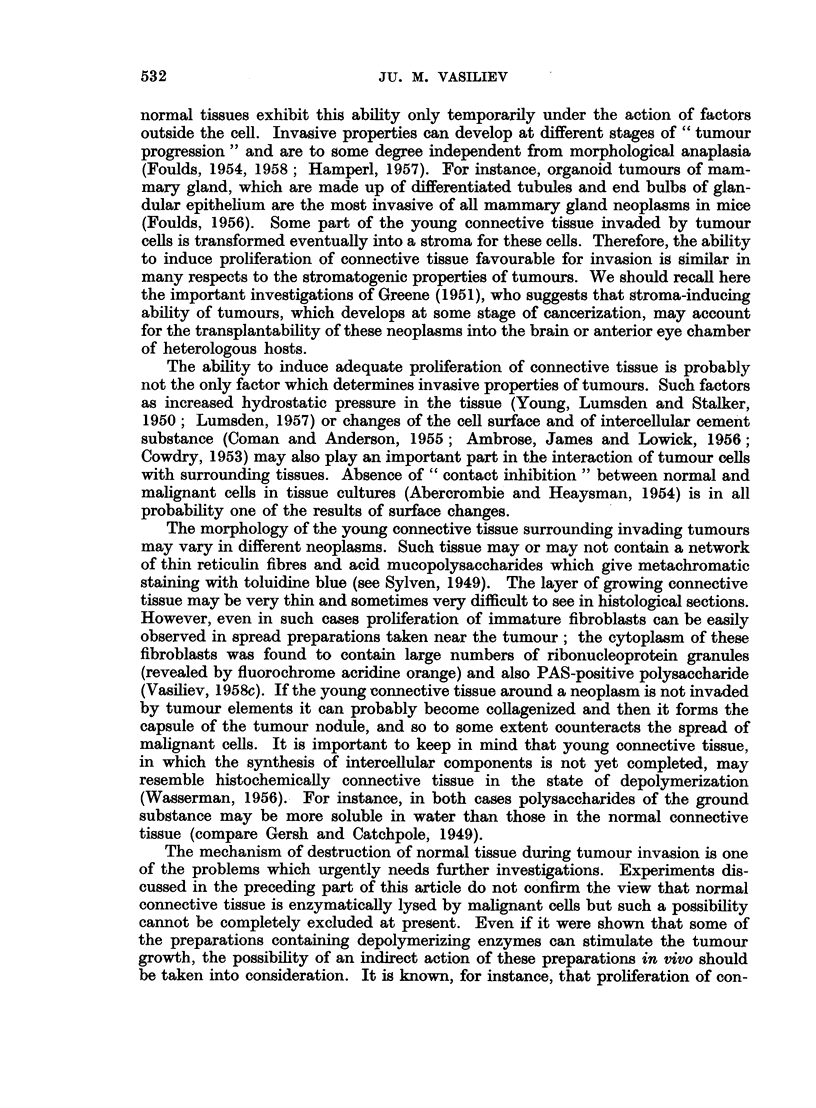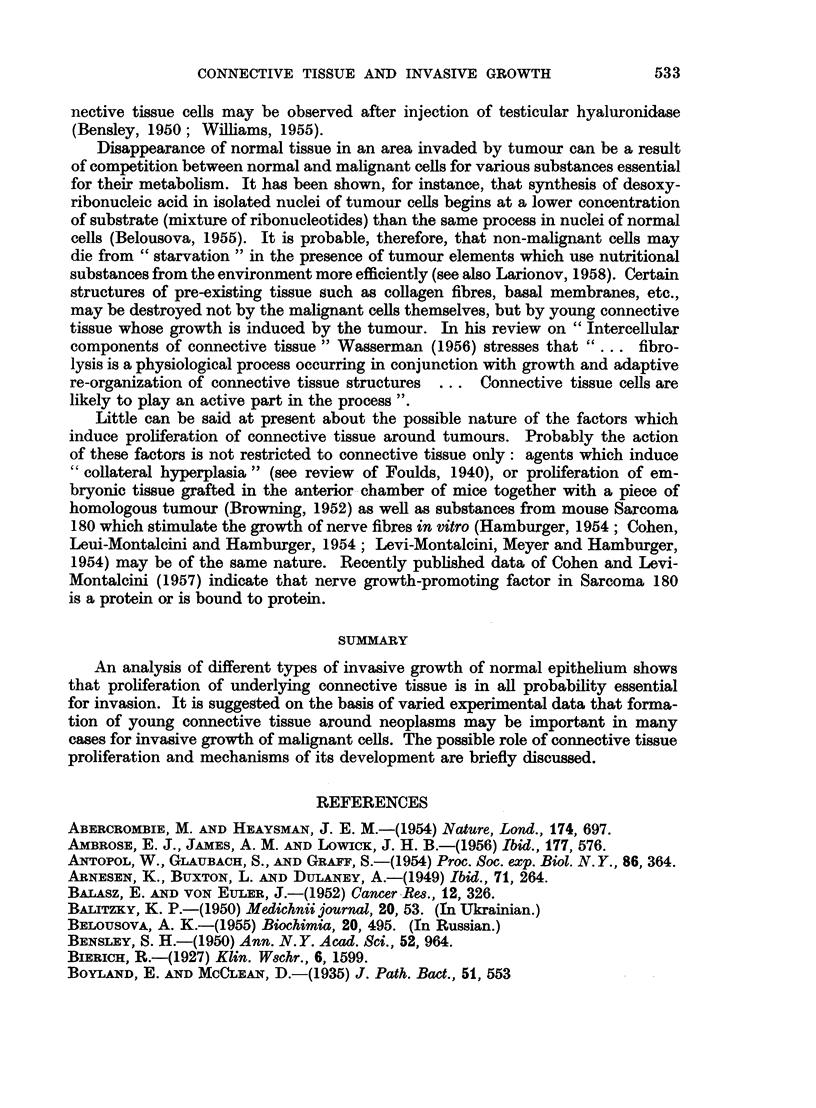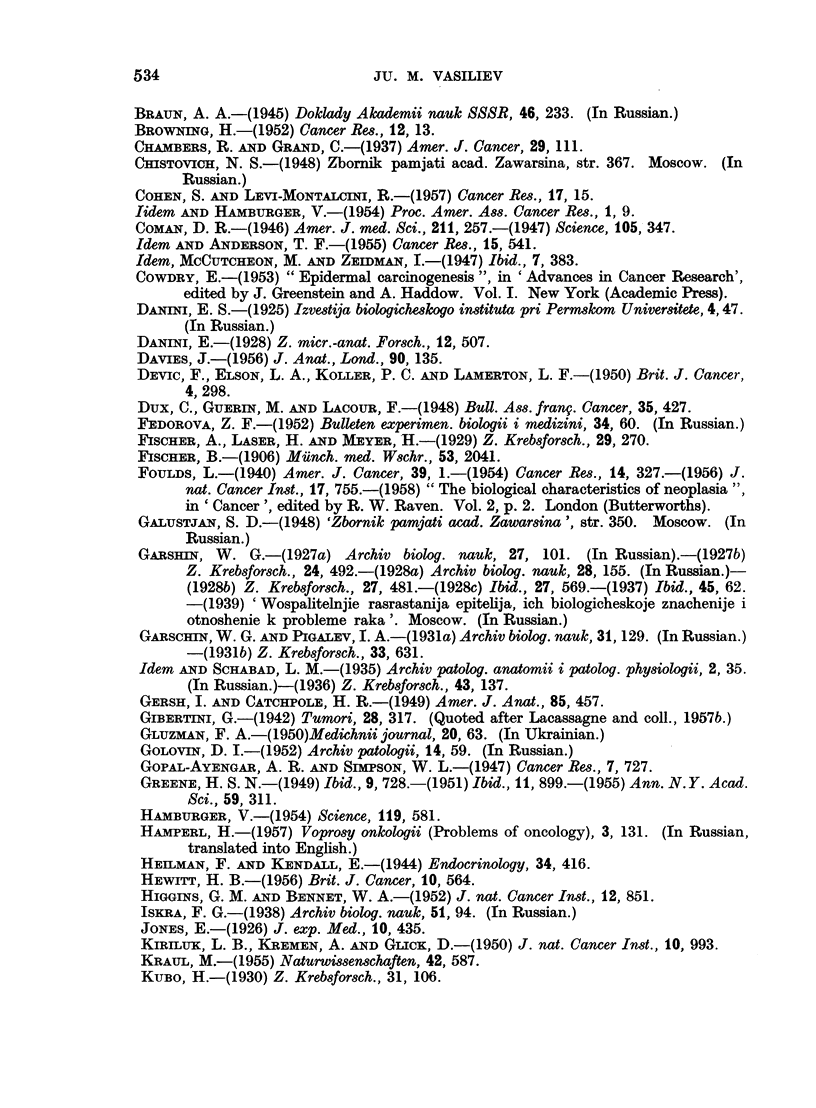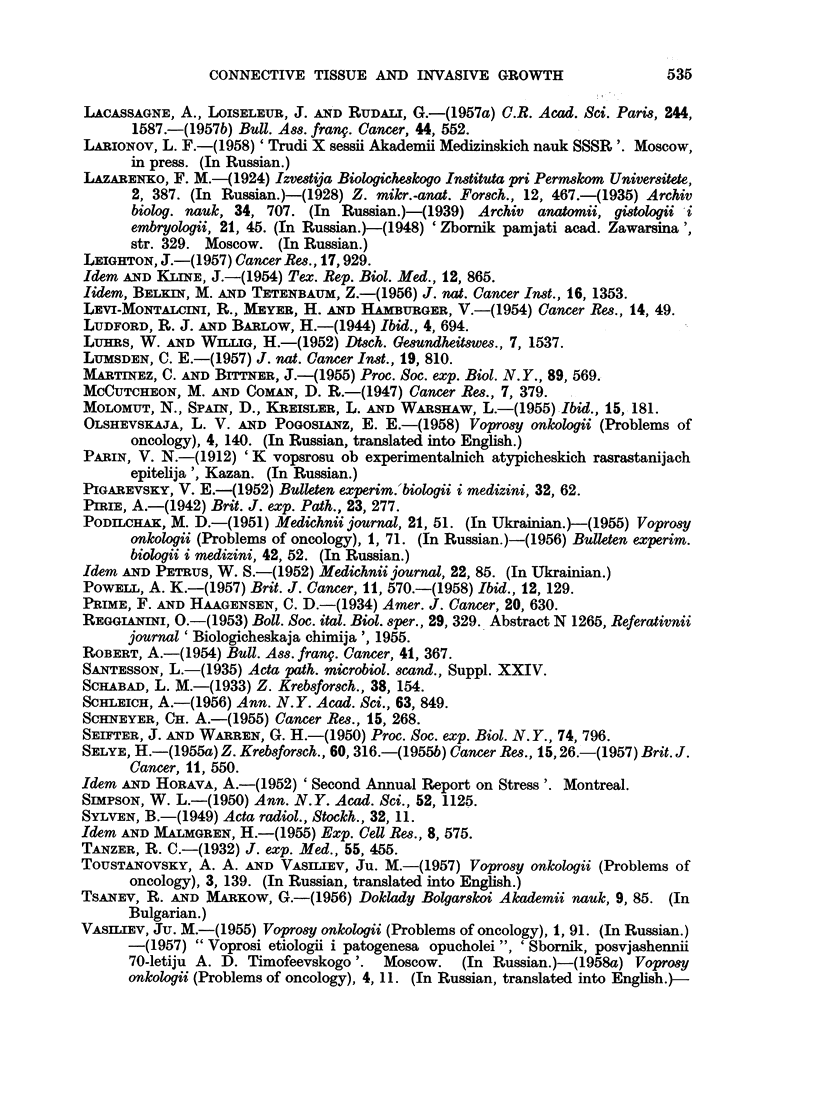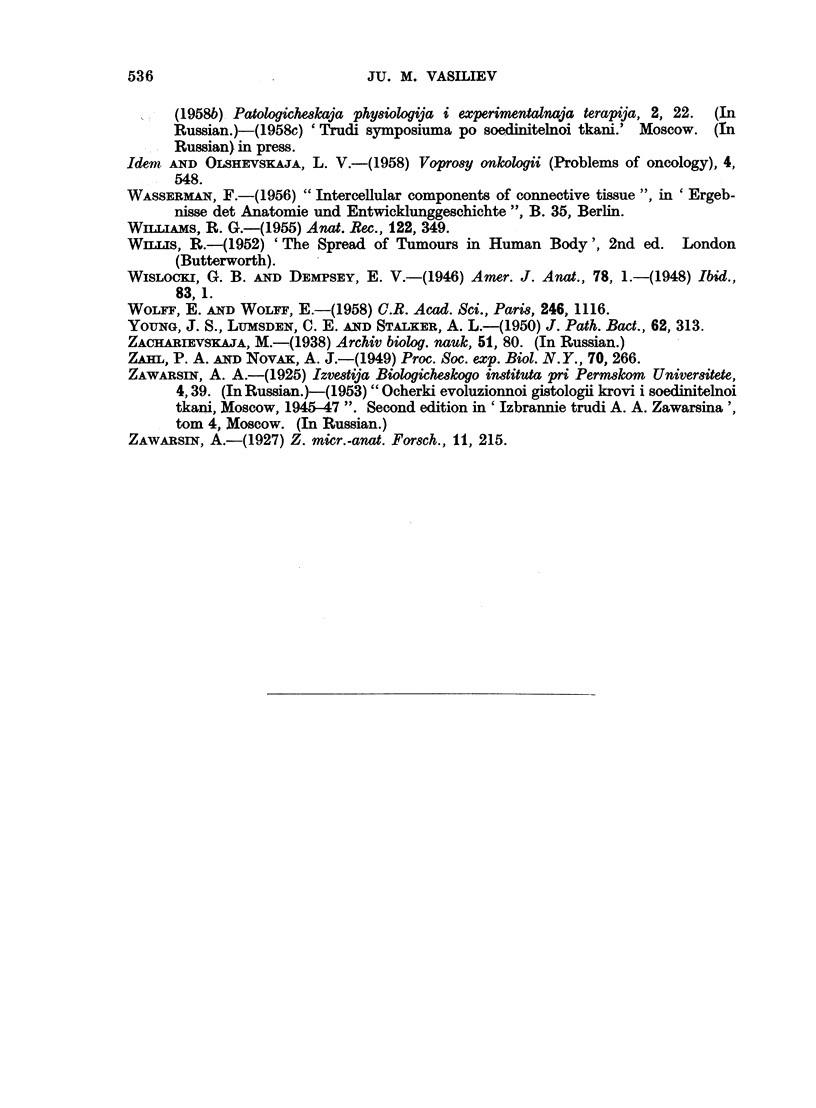# The Role of Connective Tissue Proliferation in Invasive Growth of Normal and Malignant Tissues: A Review

**DOI:** 10.1038/bjc.1958.61

**Published:** 1958-12

**Authors:** Ju. M. Vasiliev


### THE ROLE OF CONNECTIVE TISSUE PROLIFERATION IN INVASIVE GROWTH OF NORMAL AND MALIGNANT TISSUES: A REVIEW

JU. M. VASILIEV

*From the Institute of Experimental Pathology and Therapy of Cancer, Moscow, U.S.S.R.*

Received for publication 1st August, 1958

ABILITY to invade surrounding tissues is obviously one of the basic characteristics of malignant neoplasms but, in spite of this, the number of investigations dealing with this problem is not very large. Only a few reviews are devoted to this problem, among which the works of Willis (1952), Coman (1946, 1947) and Leighton (1957) should be mentioned. The main aim of this article is to discuss possible mechanisms of invasion and, in particular, to stress the possible role of connective tissue proliferation in this process.

### *I. Invasive Growth of Non-malignant Tissues*

So-called " inflammatory proliferations " of epithelium are probably the best studied examples of a process where invasive growth of the non-malignant epithelium can be observed. In 1906 Fischer described cancer-like epithelial lesions induced in the skin of rabbit ear by subcutaneously injected solutions of scarlet red. These lesions were morphologically similar to squamous carcinomas, but invariably regressed after some period of time. Various opinions have been expressed about factors which might induce such proliferation (see review of the early investigations in this field in books by Parin (1912) and Garschin (1939)). Garschin (1927*a*, *b* ; 1928*a*, *b*, *c* ; 1937 ; 1939) studied in detail the morphology of this process and came to the conclusion that invasive growth of skin epithelium was closely connected both in time and place with the inflammatory changes in subepithelial connective tissue. He supposed that lesions induced by scarlet red in the rabbit skin as well as some types of atypical epithelial proliferations observed in human pathology, belong to the class of processes designated by him as " inflammatory proliferations of epithelium ". Invasive growth of the non-malignant epithelium into the connective tissue at certain stages of inflammation (sometimes with formation of cysts around necrotic areas or around foreign bodies and with subsequent elimination of these bodies through the epithelium-covered surface) and, lastly, complete regression of the epithelial sprouts after the end of the inflammation, are, according to Garschin, characteristics of such proliferations. Inflammatory proliferations of epithelium were induced with aid of different irritative agents in various organs of laboratory mammals ; in lungs (Garschin and Pigalev, 1931*a*, *b* ; Schabad, 1933 ; Garschin and Schabad, 1935, 1936), in kidneys (Zacharievskaja, 1938), in salivary glands (Iskra, 1938), in mammary glands (Golovin, 1952) and in the skin of the rat embryos (Fedorova, 1952). Similar processes of epithelial proliferation and of invasion were observed in the experiments of Zawarsin and his collaborators, who studied the comparative morphology of experimental inflammatory reactions in invertebrate and lower vertebrate animals, namely, insects (Lazarenko, 1924, 1928), crustacea (Danini, 1925, 1928), molluscs (Zawarsin, 1925, 1927) and frogs, (Braun, 1945). In all these experiments the authors observed invasion of the young connective tissue surrounding the implanted pieces of celloidin by the epithelial sprouts growing from the adjacent epithelial structures ; in many cases formation of epithelial cysts around celloidin was observed. Zawarsin (1947) emphasizes that formation of the immature connective tissue under the basal membrane always precedes the beginning of the invasive growth of epithelium during inflammation.

Lazarenko (1935, 1939, 1948) implanted subcutaneously into rabbits and guinea-pigs pieces of celloidin mixed with the minced tissue of various organs of homologous animals (homotransplants of salivary glands, kidneys, thyroid gland and of other organs were used ; see also articles of Galustjan (1948), and of Chistovich (1948), where similar experiments respectively with thymus and with pancreas are described). Proliferation of immature fibroblasts around the implanted foreign bodies was observed in these experiments, whereas epithelial cells grew infiltratively from the transplants into this fibroblastic tissue and formed varied organoid structures. Obviously, the interrelationship between the transplanted epithelium and the surrounding connective tissue was essentially the same in these experiments as in the "inflammatory proliferation" described above.

Ectodermal proliferations associated with the mesodermal lesions that can be induced in the chorio-allantoic membrane of the chicken embryos, probably belong to the same group of processes (Olshevskaja and Pogosianz, 1958).

The invasive growth can also be observed during various processes of normal morphogenesis in embryos and in adult animals. For instance, in pregnancy, the mouse mammary gland epithelium forms the alveolar buds which grow into the surrounding connective tissue. Toustanovsky and Vasiliev (1957) have studied the changes in the stroma of mouse mammary gland during pregnancy and lactation ; various morphological and histochemical methods were used. It was observed that before the growth of epithelium began, the compact basal membrane disappeared and a net of thin fibres formed around the ducts. Later, the formation of the reticulin fibres around growing alveolar buds proceeded. Development of the young connective tissue, containing the reticulin fibres and the acid mucopolysaccharides which gave metachromatic staining with toluidine blue, were observed also in the endometrium of different mammals around the placental villi growing into the wall of pregnant uterus (Wislocki and Dempsey, 1946, 1948 ; Davies, 1956). It may be concluded that the invasive growth of the mouse mammary gland epithelium and that of the placental villi is somewhat similar to the "inflammatory proliferation" of epithelium. In all these cases formation of the "bed", consisting of the young connective tissue, precedes the epithelial invasion. The relationships of the connective tissue changes and of the epithelial growth may be different in different processes. For instance, during "inflammatory proliferation" the growth of connective tissue is induced by irritants such as scarlet red or celloidin ; the young connective tissue in its turn induces the invasive growth of the adjacent epithelium. It is possible, that in other cases the epithelium, stimulated by some endogenous agent (such as hormones), begins to secrete a substance which induced the proliferation of the connective tissues.

Finally, the proliferation of the epithelium and that of the connective tissue may be under the control of different mutually independent mechanisms; this is probably the case in placental growth. However, in all cases formation of the young connective tissue " matrix " seems to be essential for the invasive growth of the non-malignant epithelium.

### *II. The Interaction of the Growing Tumour with the Surrounding Connective Tissue*

### *Enzymes, depolymerizing the components of the connective tissue, and tumour invasion*

It has been suggested many times that the invasive growth of malignant tumours is a result of the destruction of the intercellular components of connective tissue (fibres or ground substance) caused by some agents released by cancer cells. Bierich (1927) ascribed this action to the lactic acid. When Duran-Reynals had discovered the " spreading factor", which later had been identified as hyaluronidase, many investigators made efforts to find some evidence in favour of the view that an agent of the same type was responsible for invasiveness of malignant neoplasms. Several authors have described stimulation of growth and increase of invasiveness of different transplantable tumours (Gopal-Ayengar and Simpson, 1947; Simpson, 1950; Balitzky, 1950; Podilchak, 1951; Kraul, 1955) and acceleration of the development of spontaneous mammary tumours in mice (Lacassagne, Loiseleur and Rudali, 1957*a*, *b*) after injections of the testicular extracts or of purified hyalyronidase preparations. However, many other investigators (Tanzer, 1932; Prime and Haagensen, 1934; Coman *et al.*, 1947; Arnesen, Buxton and Dulaney, 1949; Seifter and Warren, 1950; Lührs and Willig, 1952) did not observe any influence of hyaluronidase on the growth of tumour grafts or even describe inhibitory effect.

The efforts to find " spreading factors " in the tumour tissue also were not very successful in the majority of the experiments. Boyland and McClean (1935) found the " spreading factor " in aqueous extracts from tissue of rapidly growing transplantable tumours; activity of this factor was approximately proportional to the rate of growth of the neoplasm. Pirie (1942) observed weak hyaluronidase activity in 5 transplantable animal tumours. However, at the same time Gibertini (1942) did not find hyaluronidase in the majority of the extracts of 23 human tumours. McCutcheon and Coman (1947) came to the conclusion, that hyaluronidase is present only in a part of human neoplasms; activity of the enzyme in these tumours is weak. Dux, Guerin and Lacour (1948) studied 17 human tumours and 19 experimental neoplasms of animals and did not find any correlation between the malignancy of cancer and the presence of hyaluronidase. In the experiments of Gluzman (1950) " spreading factor " was found in the extracts from different grafted and methylcholanthrene-induced animal tumours. According to Podilchak and Petrus (1952) rapidly growing malignant human neoplasms contain hyaluronidase, whereas in benign tumours this enzyme is not present. However, Kiriluk, Kremen and Glick (1950) suggest that not the malignant cells themselves, but micro-organisms, contaminating neoplasm, are the source of hyaluronidase in the tumour tissue and, in fact, these authors did not find any hyaluronidase activity in tumours when special measures were taken to avoid bacterial contamination. Similar results are reported by Reggianini (1953).

Balasz and von Euler (1952) came to the conclusion that concentration of hyaluronidase is higher in necrotic parts of the Walker tumour than in the living tissue of the same tumour.

As one may see from this list of controversial findings, the opinion that hyaluronidase is the factor requisite in all cases for invasive growth is in all probability unfounded.

Some investigators think proteolytic enzymes liberated by cancer cells and not hyaluronidase play an important role in invasion. Gersh and Catchpole (1949) describe depolymerization of the PAS-positive ground substance of connective tissue by a rapidly growing transplanted tumour ; they suggest, that this depolymerization is due to the action of collagenase-like enzymes. Similar hypothesis has been put foward by Sylven (1949), who observed dissolution of the connective tissue fibres around human carcinomas. Sylven and Malmgren (1955) found that the proteolytic activity was greater in the peripheral part of the nodule of grafted tumour than in the central part of the same nodule. It is not clear, however, whether these proteolytic enzymes act *in vivo* inside the tumour cell or are liberated in the surrounding tissue.

Experiments with tissue cultures do not confirm the existence of a positive correlation between the proteolytic activity and the invasiveness of neoplasms. As the *in vitro* investigations of Santesson (1935) and later those of Leighton (1957) showed, the most malignant tumours are least proteolytic, and conversely the least malignant tumours are the most proteolytic. Leighton (1957) suggests that the capacity to make the ground substance more fluid is accompanied by a limitation of invasive spread.

We see that at present there are no facts that unequivocally confirm the view that hyaluronidase, proteases or other depolymerizing enzymes play a leading role in the invasive growth of tumours. On the contrary, some of the available data are inconsistent with such hypotheses.

*Interaction of tumour explants with connective tissue* in vitro

Some observations show that during invasive growth a complex interaction of the tumour and of the surrounding tissue takes place and that the proliferation of connective tissue is probably an important part of such interaction.

In the experiments by Leighton (Leighton and Kline, 1954 ; Leighton *et al.*, 1956 ; Leighton, 1957) the interaction of malignant human cells (HeLa carcinoma, D–189 line) and of normal tissues was studied *in vitro*, in sponge matrix tissue cultures. HeLa cells readily invaded those normal tissues of the chick embryo and of the human foetus that gave rise to a luxuriant outgrowth of connective tissue. It was shown also that malignant cells of the D–189 line and explants of normal connective tissue are attracted by, and move toward, one another. When the contact between these two tissues is established, malignant cells begin to invade connective tissue. Stimulation of the growth of connective tissue by tumour explants was observed also in the experiments of Fischer, Laser and Meyer (1929), of Santesson (1935) and of Ludford and Barlow (1944).

Wolff and Wolff (1958) successfully cultivated human malignant cells (KB strain) together with pieces of mesonephros of chick embryos. It would be important to find out whether the mesenchymal tissue of chicken embryo is the component responsible for stimulation of tumour growth in such combined cultures. It is interesting to note in this connection that as Schleich (1956) showed, the presence of normal connective tissue is necessary for survival of malignant cells of the Yoshida sarcoma *in vitro*. Powell (1957, 1958) suggests that monocytic cells contained in the explanted pieces of normal embryonic organs form *in vitro* some substances which are essential for growth of cells of the Ehrlich mouse ascites carcinoma.

*Inflammation and invasive growth of transplanted tumour cells*

Among the *in vivo* experiments confirming the important role of connective tissue reactions in the invasive spread of cancer cells, the investigations dealing with influence of inflammation on the growth of tumour transplants need discussion.*

It was shown (Devic *et al.*, 1950 ; Vasiliev, 1955) that malignant cells migrate from subcutaneous grafts of mouse and rat tumours into undifferentiated connective tissue developing around such grafts after the initial inflammatory reaction. Inflammatory reactions around transplants of heterologous tumours were not found to be different in any detail from similar initial reactions around transplants of isologous and homologous neoplasms. Malignant cells of heterografts also migrate into the young connective tissue and begin to multiply there (Vasiliev, 1958*a*). The suggestion has been put forward therefore that this connective tissue proliferation is favourable for initial spread of malignant cells from grafted tumour fragments (Devic, *et al.*, 1950 ; Vasiliev, 1955, 1958*a*). It is probable that suppression of the initial inflammation and of the following connective tissue proliferation is one of the main causes of the inhibitory action of cortisone on the growth of transplanted homologous tumours which has been observed by a number of authors (Heilman and Kendall, 1944 ; Antopol, Glaubach and Graff, 1954 ; Higgins and Bennet, 1952 ; Martinez and Bittner, 1955 ; Selye, 1955 ; Vasiliev, 1958*a*). Inhibition of the invasion of the connective tissue by carcinomatous cells from tumour fragment grafted to cortisone-treated mice have been confirmed by histological examination (Antopol, Glaubach and Graff, 1954 ; Vasiliev, 1958*a*).

It is obviously important to find out whether the stimulation of inflammatory reaction and of connective tissue proliferation may have an opposite effect, i.e. to enhance the spread of malignant cells and to increase the rate of growth of transplanted tumours. Experiments in which the influence of artificially increased inflammation on the growth of transplanted tumours were studied gave rather contradictory results : some authors (Kubo, 1930 ; Chambers and Grand, 1937 ; Pigarevsky, 1952) observed inhibition of tumour growth ; a few investigators (Molomut *et al.*, 1955 ; Hewett, 1956) did not find any effect at all, while a number of authors (see below) report that increased inflammation enhances the growth of grafts. Such contradictions may be due to differences in methods used for stimulation of inflammation and to differences in the morphology of inflammation induced by these methods. It is possible, for instance, that rapid elimination of such irritants as formic acid or allergic protein from the site of injection may account for the absence of any effect of inflammation reported by Molomut *et al.* (1955) and by Hewett (1956). Inhibition of tumour growth by infusorial earth

* It is to be stressed that only the role of initial inflammatory reactions around tumour grafts will be discussed here. Investigations dealing with other types of reactions of connective tissue cells, for instance, reactions associated with rejection of foreign tumour grafts, are obviously beyond the scope of this article.

(Kieselguhr) observed in experiments of Kubo (1930) and of Pigarevsky (1952) may be a result of the direct toxic action of this irritant on the transplanted tumour cells (see Vasiliev, 1957). Inflammation associated with extensive suppuration and tissue necrosis obviously may have a harmful effect on the tumour cells. However, it is important for our discussion that the majority of authors come to the conclusion that productive inflammation associated with proliferation of the connective tissue cells favours the establishment and growth of tumour transplants and the spread and metastasis of malignant cells.

Tsanev and Marcow (1956) in experiments with transplantable Guerin carcinoma observed that grafting of the tumour into a 6–9-day-old granulation tissue increased the frequency of metastasic growths in regional lymph glands and also shortened the average latent period of their appearance and the survival time of tumour-bearing rats. According to Podilchak (1955) Brown-Pearce rabbit carcinoma metastasized more often to the spleen if a focus of chronic inflammation had been induced in that organ. In experiments described in another communication, Podilchak (1956) observed an increase of the frequency of metastasis of the Brown-Pearce tumour to the stomach after the induction of chronic inflammation in the organ. Zahl and Novac (1949) reported that mechanical injury of the transplantation site increased the rate of growth of transplantable mouse sarcoma. In the experiments of Jones (1926) transplantable mouse mammary gland tumour of DBA strain could be transplanted successfully to otherwise resistant C57Bl mice if a local irritant (a piece of sterile flannel) was introduced subcutaneously together with the tumour graft.

In a number of recent communications a new technique for study of inflammation—so-called " air-pouch technique " described by Selye and Horava (1952) has been used. In the experiments of Selye (1955) Walker carcinosarcoma was grafted into the " air pouches " of rats ; combined treatment of animals with small doses of cortisol and with $NH_4Cl$, which suppressed accumulation of inflammatory exudate in adrenalectomized rats also inhibited the growth of the transplanted tumour. Robert (1954) reported that grafting of transplantable mouse mammary tumour into an " air-pouch " made on the neck of a mouse resulted in stimulation of growth of the graft as compared with routine subcutaneous transplantation. On the contrary, Hewett (1956) did not find any difference between the growth rates of mouse Sarcoma 37 transplanted subcutaneously and in an " air-pouch ". It is possible, however, that in these experiments the quantity of the tumour tissue injected to each animal was too small and did not induce a sufficient degree of inflammation in the " air-pouch ". This suggestion is confirmed by the fact that the accumulation of exudate in the " air-pouches " with grafted tumours, which had been observed by other investigators, did not occur in Hewett's experiments.

Vasiliev (1957) observed that after injection of tumour suspension in an " air-pouch " the initial inflammation was more pronounced and the young connective tissue formed after such inflammation covered a much wider area than after subcutaneous grafting. Striking stimulation of growth of a number of isologous and strain-non-specific homologous grafts of mouse and rat tumours transplanted into " air-pouches " was observed in these experiments. Mouse Sarcoma 180 grafted into " air-pouches " of weanling rats grew there much more rapidly than when transplanted subcutaneously, so that the majority of animals died with huge tumours in " air-pouches " 6–8 days after grafting ; it was possible to propagate mouse Sarcoma 180 serially in the " air-pouches " of weanling rats in a number of passages without cortisone treatment of the heterologous hosts. If animals did not die from tumour growth in the first 10 days of the experiment, heterotransplants in the " air-pouches " regressed at the same time as in control animals with subcutaneous grafts of the same mouse tumour : that is from 11 to 13 days after transplantation. Other mouse tumours used in these experiments (RSM strain of mammary gland carcinoma and hepatoma XXII) regressed in untreated weanling rats earlier than Sarcoma 180—at 7–8 days after grafting. In cortisone-treated rats these tumours grew for a long period ; the final weight of these heterologous tumours transplanted into " air-pouches " was 3–4 times as high as in control rats with subcutaneous transplants, which received similar cortisone treatment.

Selye (1957) grafted suspension of the Walker tumour into " air-pouches " made on the backs of rats ; simultaneously croton oil was injected into " air-pouches " of some of the rats. Selye came to the conclusion that the stimulation of inflammation by croton oil enhances the growth of transplants.

Results of the experiments quoted above show that stimulation of inflammation and of connective tissue proliferation around grafts of homologous and heterologous tumours can in many cases facilitate the establishment and enhance the growth of such grafts. These data are in good agreement with the idea of the importance of connective tissue proliferation for invasive growth of malignant cells.

*Influence of embryonic tissue on the growth of tumour transplants*

Experiments of another type, which deserve discussion here, are those dealing with the effect of embryonic tissue on the growth of tumour transplants. Greene (1949, 1955) reported that in experiments with transplantation of animal and human tumours into the anterior eye chamber or brain of heterologous hosts the percentage of the positive takes increased if pieces of embryonic tissue were added to the graft. Greene suggested that embryonic tissue either evoked stromal reactions of the host or served as primary stroma for the transplanted tumour. As Schneyer (1955) showed, the growth rate of a transplantable mouse mammary carcinoma significantly increased if the tumour suspension had been mixed with isologous embryonic tissue before grafting.

In the experiments of Vasiliev (1958*b*) and of Vasiliev and Olshevskaja (1958) suspensions of embryonic tissues were mixed *in vitro* with the suspensions of tumour cells immediately before transplantation. It was shown that such embryonic tissue suspensions significantly accelerated the growth of homografts of strain-non-specific tumours (mouse Sarcoma 180 and rat Sarcoma 45) and also that of heterografts of mouse Sarcoma 180 in weanling rats. The embryonic tissues, whose species specificity corresponded to that of the host, were the most active, for instance, mouse embryonic tissues were active in experiments with homografts of mouse Sarcoma 180 and rat embryonic tissues in experiments with the same tumour grafted into rats. Embryonic tissue lost its activity after heating to 60° C. or freezing to −40° C. Different homologous and heterologous tissues of adult animals had no growth-accelerating properties. It was suggested that stimulation of tumour growth is due to the presence of living embryonic cells at the site of transplantation. Inactivity of heterologous embryonic tissues may then be regarded as a result of their rapid destruction in the host's organism.

Accumulation of a large number of immature fibroblasts at the site of the injection of suspensions of homologous embryonic tissues had been observed in rats by Vasiliev and Olshevskaja (1958). It is not clear at present whether these cells migrate from the grafted pieces of embryonic organs or are host elements which begin to multiply under the influence of the transplant. It is tempting to suggest that the presence of such fibroblasts is the factor responsible for stimulation of growth of the transplanted tumour. In any case it is obvious that combined transplants consisting of the embryonic and malignant tissue are similar in many respects to combined explants studied in the experiments of Leighton, Wolff and others (see above). In both cases embryonic tissue probably may serve as an artificially provided " matrix " for tumour cells, as an environment favourable for the multiplication and invasive spread of these cells. Thus, the role of embryonic tissue in these experiments is, probably, the same as that of undifferentiated connective tissue developing around a growing tumour.

### DISCUSSION

Several suggestions may be put forward on the basis of the facts reviewed above. It seems probably that in many cases proliferation of connective tissue plays an important part in invasive growth of tumours. Possibly such proliferation is not requisite for all processes of invasion ; for instance, it is probably unnecessary for invasion of tissues by white blood cells as well as by their malignant counterparts. However, for many types of tumours the formation of undifferentiated connective tissue seems to be an essential part of the mechanism of invasion. Such connective tissue can have several functions during invasion. It can form a network of thin microscopic and submicroscopic fibres serving as matrix which gives mechanical support for tumour cells. During " inflammatory proliferation " growing connective tissue somehow attracts the adjacent epithelium. It may be suggested that some hypothetical substances liberated by young connective tissue surrounding a tumour also attract the malignant cells which then begin to invade this tissue ; *in vitro* observations by Leighton (see above) give some support to this idea. Finally, young connective tissue can create a uniform chemical environment favourable for tumour cells. We have mentioned already experiments which show that *in vitro* normal tissue explants liberate into the medium some substances essential for survival of the ascites tumour cells. It is possible that connective tissue can form such substances *in vivo*.

Close association of invasive spread and of connective tissue proliferation is not only a characteristic of malignant tumours ; as we tried to show in the first part of this review, similar relationships can be observed during invasive growth of normal tissues. Investigations by Zawarsin and his collaborators make clear that invasive growth of non-malignant epithelium into young connective tissue may occur in invertebrate animals. Thus, such type of morphogenetic reaction has been developed at a relatively early stage of the evolutionary process. The basic mechanisms of invasion are probably the same for normal cells and for malignant neoplasms, but in cancer tissue these mechanisms are at work for indefinitely long periods whereas in normal tissues they start and cease work at a definite time, for instance, at a certain stage of inflammation or under the influence of some endocrine change. It is possible, therefore, that cancer cells acquire an intrinsic ability to evoke proliferation of connective tissue, whereas normal tissues exhibit this ability only temporarily under the action of factors outside the cell. Invasive properties can develop at different stages of " tumour progression " and are to some degree independent from morphological anaplasia (Foulds, 1954, 1958 ; Hamperl, 1957). For instance, organoid tumours of mammary gland, which are made up of differentiated tubules and end bulbs of glandular epithelium are the most invasive of all mammary gland neoplasms in mice (Foulds, 1956). Some part of the young connective tissue invaded by tumour cells is transformed eventually into a stroma for these cells. Therefore, the ability to induce proliferation of connective tissue favourable for invasion is similar in many respects to the stromatogenic properties of tumours. We should recall here the important investigations of Greene (1951), who suggests that stroma-inducing ability of tumours, which develops at some stage of cancerization, may account for the transplantability of these neoplasms into the brain or anterior eye chamber of heterologous hosts.

The ability to induce adequate proliferation of connective tissue is probably not the only factor which determines invasive properties of tumours. Such factors as increased hydrostatic pressure in the tissue (Young, Lumsden and Stalker, 1950 ; Lumsden, 1957) or changes of the cell surface and of intercellular cement substance (Coman and Anderson, 1955 ; Ambrose, James and Lowick, 1956 ; Cowdry, 1953) may also play an important part in the interaction of tumour cells with surrounding tissues. Absence of " contact inhibition " between normal and malignant cells in tissue cultures (Abercrombie and Heaysman, 1954) is in all probability one of the results of surface changes.

The morphology of the young connective tissue surrounding invading tumours may vary in different neoplasms. Such tissue may or may not contain a network of thin reticulin fibres and acid mucopolysaccharides which give metachromatic staining with toluidine blue (see Sylven, 1949). The layer of growing connective tissue may be very thin and sometimes very difficult to see in histological sections. However, even in such cases proliferation of immature fibroblasts can be easily observed in spread preparations taken near the tumour ; the cytoplasm of these fibroblasts was found to contain large numbers of ribonucleoprotein granules (revealed by fluorochrome acridine orange) and also PAS-positive polysaccharide (Vasiliev, 1958*c*). If the young connective tissue around a neoplasm is not invaded by tumour elements it can probably become collagenized and then it forms the capsule of the tumour nodule, and so to some extent counteracts the spread of malignant cells. It is important to keep in mind that young connective tissue, in which the synthesis of intercellular components is not yet completed, may resemble histochemically connective tissue in the state of depolymerization (Wasserman, 1956). For instance, in both cases polysaccharides of the ground substance may be more soluble in water than those in the normal connective tissue (compare Gersh and Catchpole, 1949).

The mechanism of destruction of normal tissue during tumour invasion is one of the problems which urgently needs further investigations. Experiments discussed in the preceding part of this article do not confirm the view that normal connective tissue is enzymatically lysed by malignant cells but such a possibility cannot be completely excluded at present. Even if it were shown that some of the preparations containing depolymerizing enzymes can stimulate the tumour growth, the possibility of an indirect action of these preparations *in vivo* should be taken into consideration. It is known, for instance, that proliferation of connective tissue cells may be observed after injection of testicular hyaluronidase (Bensley, 1950; Williams, 1955).

Disappearance of normal tissue in an area invaded by tumour can be a result of competition between normal and malignant cells for various substances essential for their metabolism. It has been shown, for instance, that synthesis of desoxyribonucleic acid in isolated nuclei of tumour cells begins at a lower concentration of substrate (mixture of ribonucleotides) than the same process in nuclei of normal cells (Belousova, 1955). It is probable, therefore, that non-malignant cells may die from " starvation " in the presence of tumour elements which use nutritional substances from the environment more efficiently (see also Larionov, 1958). Certain structures of pre-existing tissue such as collagen fibres, basal membranes, etc., may be destroyed not by the malignant cells themselves, but by young connective tissue whose growth is induced by the tumour. In his review on " Intercellular components of connective tissue " Wasserman (1956) stresses that " ... fibrolysis is a physiological process occurring in conjunction with growth and adaptive re-organization of connective tissue structures ... Connective tissue cells are likely to play an active part in the process ".

Little can be said at present about the possible nature of the factors which induce proliferation of connective tissue around tumours. Probably the action of these factors is not restricted to connective tissue only: agents which induce " collateral hyperplasia " (see review of Foulds, 1940), or proliferation of embryonic tissue grafted in the anterior chamber of mice together with a piece of homologous tumour (Browning, 1952) as well as substances from mouse Sarcoma 180 which stimulate the growth of nerve fibres *in vitro* (Hamburger, 1954; Cohen, Leui-Montalcini and Hamburger, 1954; Levi-Montalcini, Meyer and Hamburger, 1954) may be of the same nature. Recently published data of Cohen and Levi-Montalcini (1957) indicate that nerve growth-promoting factor in Sarcoma 180 is a protein or is bound to protein.

### SUMMARY

An analysis of different types of invasive growth of normal epithelium shows that proliferation of underlying connective tissue is in all probability essential for invasion. It is suggested on the basis of varied experimental data that formation of young connective tissue around neoplasms may be important in many cases for invasive growth of malignant cells. The possible role of connective tissue proliferation and mechanisms of its development are briefly discussed.

### REFERENCES

Abercrombie, M. and Heaysman, J. E. M.—(1954) *Nature, Lond.*, **174**, 697.
Ambrose, E. J., James, A. M. and Lowick, J. H. B.—(1956) *Ibid.*, **177**, 576.
Antopol, W., Glaubach, S., and Graff, S.—(1954) *Proc. Soc. exp. Biol. N.Y.*, **86**, 364.
Arnesen, K., Buxton, L. and Dulaney, A.—(1949) *Ibid.*, **71**, 264.
Balasz, E. and von Euler, J.—(1952) *Cancer Res.*, **12**, 326.
Balitzky, K. P.—(1950) *Medichnii journal*, **20**, 53. (In Ukrainian.)
Belousova, A. K.—(1955) *Biochimia*, **20**, 495. (In Russian.)
Bensley, S. H.—(1950) *Ann. N.Y. Acad. Sci.*, **52**, 964.
Bierich, R.—(1927) *Klin. Wschr.*, **6**, 1599.
Boyland, E. and McClean, D.—(1935) *J. Path. Bact.*, **51**, 553

BRAUN, A. A.—(1945) *Doklady Akademii nauk SSSR*, **46**, 233. (In Russian.)
BROWNING, H.—(1952) *Cancer Res.*, **12**, 13.
CHAMBERS, R. AND GRAND, C.—(1937) *Amer. J. Cancer*, **29**, 111.
CHISTOVICH, N. S.—(1948) Zbornik pamjati acad. Zawarsina, str. 367. Moscow. (In Russian.)
COHEN, S. AND LEVI-MONTALCINI, R.—(1957) *Cancer Res.*, **17**, 15.
*Iidem* AND HAMBURGER, V.—(1954) *Proc. Amer. Ass. Cancer Res.*, **1**, 9.
COMAN, D. R.—(1946) *Amer. J. med. Sci.*, **211**, 257.—(1947) *Science*, **105**, 347.
*Idem* AND ANDERSON, T. F.—(1955) *Cancer Res.*, **15**, 541.
*Idem*, MCCUTCHEON, M. AND ZEIDMAN, I.—(1947) *Ibid.*, **7**, 383.
COWDRY, E.—(1953) " Epidermal carcinogenesis ", in ' Advances in Cancer Research', edited by J. Greenstein and A. Haddow. Vol. I. New York (Academic Press).
DANINI, E. S.—(1925) *Izvestija biologicheskogo instituta pri Permskom Universitete*, **4**, 47. (In Russian.)
DANINI, E.—(1928) *Z. micr.-anat. Forsch.*, **12**, 507.
DAVIES, J.—(1956) *J. Anat., Lond.*, **90**, 135.
DEVIC, F., ELSON, L. A., KOLLER, P. C. AND LAMERTON, L. F.—(1950) *Brit. J. Cancer*, **4**, 298.
DUX, C., GUERIN, M. AND LACOUR, F.—(1948) *Bull. Ass. franç. Cancer*, **35**, 427.
FEDOROVA, Z. F.—(1952) *Bulleten experimen. biologii i medizini*, **34**, 60. (In Russian.)
FISCHER, A., LASER, H. AND MEYER, H.—(1929) *Z. Krebsforsch.*, **29**, 270.
FISCHER, B.—(1906) *Münch. med. Wschr.*, **53**, 2041.
FOULDS, L.—(1940) *Amer. J. Cancer*, **39**, 1.—(1954) *Cancer Res.*, **14**, 327.—(1956) *J. nat. Cancer Inst.*, **17**, 755.—(1958) " The biological characteristics of neoplasia ", in ' Cancer ', edited by R. W. Raven. Vol. 2, p. 2. London (Butterworths).
GALUSTJAN, S. D.—(1948) '*Zbornik pamjati acad. Zawarsina* ', str. 350. Moscow. (In Russian.)
GARSHIN, W. G.—(1927*a*) *Archiv biolog. nauk*, **27**, 101. (In Russian).—(1927*b*) *Z. Krebsforsch.*, **24**, 492.—(1928*a*) *Archiv biolog. nauk*, **28**, 155. (In Russian.)—(1928*b*) *Z. Krebsforsch.*, **27**, 481.—(1928*c*) *Ibid.*, **27**, 569.—(1937) *Ibid.*, **45**, 62.—(1939) ' Wospalitelnjie rasrastanija epitelija, ich biologicheskoje znachenije i otnoshenie k probleme raka '. Moscow. (In Russian.)
GARSCHIN, W. G. AND PIGALEV, I. A.—(1931*a*) *Archiv biolog. nauk*, **31**, 129. (In Russian.)—(1931*b*) *Z. Krebsforsch.*, **33**, 631.
*Idem* AND SCHABAD, L. M.—(1935) *Archiv patolog. anatomii i patolog. physiologii*, **2**, 35. (In Russian.)—(1936) *Z. Krebsforsch.*, **43**, 137.
GERSH, I. AND CATCHPOLE, H. R.—(1949) *Amer. J. Anat.*, **85**, 457.
GIBERTINI, G.—(1942) *Tumori*, **28**, 317. (Quoted after Lacassagne and coll., 1957*b*.)
GLUZMAN, F. A.—(1950)*Medichnii journal*, **20**, 63. (In Ukrainian.)
GOLOVIN, D. I.—(1952) *Archiv patologii*, **14**, 59. (In Russian.)
GOPAL-AYENGAR, A. R. AND SIMPSON, W. L.—(1947) *Cancer Res.*, **7**, 727.
GREENE, H. S. N.—(1949) *Ibid.*, **9**, 728.—(1951) *Ibid.*, **11**, 899.—(1955) *Ann. N.Y. Acad. Sci.*, **59**, 311.
HAMBURGER, V.—(1954) *Science*, **119**, 581.
HAMPERL, H.—(1957) *Voprosy onkologii* (Problems of oncology), **3**, 131. (In Russian, translated into English.)
HEILMAN, F. AND KENDALL, E.—(1944) *Endocrinology*, **34**, 416.
HEWITT, H. B.—(1956) *Brit. J. Cancer*, **10**, 564.
HIGGINS, G. M. AND BENNET, W. A.—(1952) *J. nat. Cancer Inst.*, **12**, 851.
ISKRA, F. G.—(1938) *Archiv biolog. nauk*, **51**, 94. (In Russian.)
JONES, E.—(1926) *J. exp. Med.*, **10**, 435.
KIRILUK, L. B., KREMEN, A. AND GLICK, D.—(1950) *J. nat. Cancer Inst.*, **10**, 993.
KRAUL, M.—(1955) *Naturwissenschaften*, **42**, 587.
KUBO, H.—(1930) *Z. Krebsforsch.*, 31, 106.

LACASSAGNE, A., LOISELEUR, J. AND RUDALI, G.—(1957*a*) *C.R. Acad. Sci. Paris*, **244**, 1587.—(1957*b*) *Bull. Ass. franç. Cancer*, **44**, 552.
LARIONOV, L. F.—(1958) ' Trudi X sessii Akademii Medizinskich nauk SSSR '. Moscow, in press. (In Russian.)
LAZARENKO, F. M.—(1924) *Izvestija Biologicheskogo Instituta pri Permskom Universitete*, 2, 387. (In Russian.)—(1928) *Z. mikr.-anat. Forsch.*, 12, 467.—(1935) *Archiv biolog. nauk*, **34**, 707. (In Russian.)—(1939) *Archiv anatomii, gistologii i embryologii*, **21**, 45. (In Russian.)—(1948) ' Zbornik pamjati acad. Zawarsina ', str. 329. Moscow. (In Russian.)
LEIGHTON, J.—(1957) *Cancer Res.*, **17**, 929.
*Idem* AND KLINE, J.—(1954) *Tex. Rep. Biol. Med.*, **12**, 865.
*Iidem*, BELKIN, M. AND TETENBAUM, Z.—(1956) *J. nat. Cancer Inst.*, **16**, 1353.
LEVI-MONTALCINI, R., MEYER, H. AND HAMBURGER, V.—(1954) *Cancer Res.*, **14**, 49.
LUDFORD, R. J. AND BARLOW, H.—(1944) *Ibid.*, **4**, 694.
LUHRS, W. AND WILLIG, H.—(1952) *Dtsch. Gesundheitswes.*, **7**, 1537.
LUMSDEN, C. E.—(1957) *J. nat. Cancer Inst.*, **19**, 810.
MARTINEZ, C. AND BITTNER, J.—(1955) *Proc. Soc. exp. Biol. N.Y.*, **89**, 569.
MCCUTCHEON, M. AND COMAN, D. R.—(1947) *Cancer Res.*, **7**, 379.
MOLOMUT, N., SPAIN, D., KREISLER, L. AND WARSHAW, L.—(1955) *Ibid.*, **15**, 181.
OLSHEVSKAJA, L. V. AND POGOSIANZ, E. E.—(1958) *Voprosy onkologii* (Problems of oncology), **4**, 140. (In Russian, translated into English.)
PARIN, V. N.—(1912) ' K vopsrosu ob experimentalnich atypicheskich rasrastanijach epitelija ', Kazan. (In Russian.)
PIGAREVSKY, V. E.—(1952) *Bulleten experim. biologii i medizini*, **32**, 62.
PIRIE, A.—(1942) *Brit. J. exp. Path.*, **23**, 277.
PODILCHAK, M. D.—(1951) *Medichnii journal*, **21**, 51. (In Ukrainian.)—(1955) *Voprosy onkologii* (Problems of oncology), **1**, 71. (In Russian.)—(1956) *Bulleten experim. biologii i medizini*, **42**, 52. (In Russian.)
*Idem* AND PETRUS, W. S.—(1952) *Medichnii journal*, **22**, 85. (In Ukrainian.)
POWELL, A. K.—(1957) *Brit. J. Cancer*, **11**, 570.—(1958) *Ibid.*, **12**, 129.
PRIME, F. AND HAAGENSEN, C. D.—(1934) *Amer. J. Cancer*, **20**, 630.
REGGIANINI, O.—(1953) *Boll. Soc. ital. Biol. sper.*, **29**, 329. Abstract N 1265, *Referativnii journal* ' Biologicheskaja chimija ', 1955.
ROBERT, A.—(1954) *Bull. Ass. franç. Cancer*, **41**, 367.
SANTESSON, L.—(1935) *Acta path. microbiol. scand.*, Suppl. XXIV.
SCHABAD, L. M.—(1933) *Z. Krebsforsch.*, **38**, 154.
SCHLEICH, A.—(1956) *Ann. N.Y. Acad. Sci.*, **63**, 849.
SCHNEYER, CH. A.—(1955) *Cancer Res.*, **15**, 268.
SEIFTER, J. AND WARREN, G. H.—(1950) *Proc. Soc. exp. Biol. N.Y.*, **74**, 796.
SELYE, H.—(1955*a*) *Z. Krebsforsch.*, **60**, 316.—(1955*b*) *Cancer Res.*, **15**, 26.—(1957) *Brit. J. Cancer*, **11**, 550.
*Idem* AND HORAVA, A.—(1952) ' Second Annual Report on Stress '. Montreal.
SIMPSON, W. L.—(1950) *Ann. N.Y. Acad. Sci.*, **52**, 1125.
SYLVEN, B.—(1949) *Acta radiol., Stockh.*, **32**, 11.
*Idem* AND MALMGREN, H.—(1955) *Exp. Cell Res.*, **8**, 575.
TANZER, R. C.—(1932) *J. exp. Med.*, **55**, 455.
TOUSTANOVSKY, A. A. AND VASILIEV, JU. M.—(1957) *Voprosy onkologii* (Problems of oncology), **3**, 139. (In Russian, translated into English.)
TSANEV, R. AND MARKOW, G.—(1956) *Doklady Bolgarskoi Akademii nauk*, **9**, 85. (In Bulgarian.)
VASILIEV, JU. M.—(1955) *Voprosy onkologii* (Problems of oncology), **1**, 91. (In Russian.) —(1957) " Voprosi etiologii i patogenesa opucholei ", ' Sbornik, posvjashennii 70-letiju A. D. Timofeevskogo '. Moscow. (In Russian.)—(1958*a*) *Voprosy onkologii* (Problems of oncology), **4**, 11. (In Russian, translated into English.)—

(1958*b*) *Patologicheskaja physiologija i experimentalnaja terapija*, **2**, 22. (In Russian.)—(1958*c*) 'Trudi symposiuma po soedinitelnoi tkani.' Moscow. (In Russian) in press.
*Idem* AND OLSHEVSKAJA, L. V.—(1958) *Voprosy onkologii* (Problems of oncology), **4**, 548.
WASSERMAN, F.—(1956) " Intercellular components of connective tissue ", in ' Ergebnisse det Anatomie und Entwicklunggeschichte ", B. 35, Berlin.
WILLIAMS, R. G.—(1955) *Anat. Rec.*, **122**, 349.
WILLIS, R.—(1952) 'The Spread of Tumours in Human Body', 2nd ed. London (Butterworth).
WISLOCKI, G. B. AND DEMPSEY, E. V.—(1946) *Amer. J. Anat.*, **78**, 1.—(1948) *Ibid.*, **83**, 1.
WOLFF, E. AND WOLFF, E.—(1958) *C.R. Acad. Sci., Paris*, **246**, 1116.
YOUNG, J. S., LUMSDEN, C. E. AND STALKER, A. L.—(1950) *J. Path. Bact.*, **62**, 313.
ZACHARIEVSKAJA, M.—(1938) *Archiv biolog. nauk*, **51**, 80. (In Russian.)
ZAHL, P. A. AND NOVAK, A. J.—(1949) *Proc. Soc. exp. Biol. N.Y.*, **70**, 266.
ZAWARSIN, A. A.—(1925) *Izvestija Biologicheskogo instituta pri Permskom Universitete*, **4**, 39. (In Russian.)—(1953) "Ocherki evoluzionnoi gistologii krovi i soedinitelnoi tkani, Moscow, 1945–47 ". Second edition in ' Izbrannie trudi A. A. Zawarsina ', tom 4, Moscow. (In Russian.)
ZAWARSIN, A.—(1927) *Z. micr.-anat. Forsch.*, **11**, 215.